# Autophagic Activity in Peripheral Blood Mononuclear Cells of Critically Ill Patients With and Without Intensive Care Unit-Acquired Weakness: A Cross-Sectional Observational Study

**DOI:** 10.7759/cureus.82706

**Published:** 2025-04-21

**Authors:** Yuki Iida, Ayato Shinohara, Tomoyuki Nakamura, Naohide Kuriyama, Osamu Nishida

**Affiliations:** 1 Department of Medical Sciences, Aichi Shukutoku University, Nagakute, JPN; 2 Department of Anesthesiology and Critical Care Medicine, Fujita Health University, Toyoake, JPN; 3 Department of Rehabilitation, Fujita Health University Hospital, Toyoake, JPN

**Keywords:** autophagy, critically ill patients, icu-acquired weakness, immunity, peripheral blood mononuclear cells

## Abstract

Introduction

This study aimed to compare autophagic activity in peripheral blood mononuclear cells (PBMCs) between intensive care unit-acquired weakness (ICU-AW) and non-ICU-AW patients, and to evaluate phase-specific differences and their associations with immune and inflammatory profiles.

Methods

This single-center, cross-sectional observational study included 42 patients who required mechanical ventilation for more than 48 hours between April 2020 and March 2022. PBMCs were collected within 48 hours of ICU admission (early phase) and on day 7 (late phase). Autophagic activity, assessed by mean fluorescence intensity (MFI), was evaluated via flow cytometry using DAPGreen (Dojindo, Kumamoto, Japan). ICU-AW was diagnosed based on a Medical Research Council sum score of less than 48 points.

Results

Among the 42 patients, 14 (33.3%) developed ICU-AW. PBMCs from ICU-AW patients demonstrated significantly lower autophagic activity in the early phase compared to non-ICU-AW patients (non-ICU-AW vs. ICU-AW patients: MFI of granulocytes, 30.9 (22.6, 51.7) vs. 20.4 (18.0, 22.6), p < 0.001; and lymphocytes, 94.6 (64.9, 123.0) vs. 65.2 (58.0, 77.5), p = 0.011). In contrast, excessive autophagic activity was observed in some ICU-AW cases during the late phase (MFI of granulocytes, 21.0 (17.9, 22.9) vs. 33.8 (22.9, 56.0), p < 0.001; and lymphocytes, 67.5 (54.4, 93.5) vs. 106.2 (64.6, 124.5), p = 0.012). The proportion of monocytes was also significantly reduced in the ICU-AW group. These findings suggest that impaired early-phase autophagy may contribute to ICU-AW pathogenesis, whereas delayed overactivation could be associated with persistent inflammation and impaired muscle recovery.

Conclusion

Autophagic activity in PBMCs exhibited temporal alterations in patients with ICU-AW. These findings suggest a potential association between dysregulated autophagy and muscle dysfunction in critically ill patients. Further research is needed to explore whether modulation of autophagy could inform future preventive strategies.

## Introduction

Advancements in intensive care medicine have markedly improved the survival of critically ill patients. However, many survivors continue to experience persistent and profound muscle weakness following intensive care unit (ICU) discharge. This condition, characterized by generalized muscle weakness and functional impairment, is known as intensive care unit-acquired weakness (ICU-AW) [1]. ICU-AW has been reported to affect 49%-77% of patients requiring prolonged ICU care (>7 days), and 25%-33% of those requiring mechanical ventilation for more than four days. Its high prevalence has become a major clinical concern, given its association with long-term reductions in activities of daily living, diminished social participation, and lower health-related quality of life [2]. Consequently, the prevention of ICU-AW is an urgent issue in the field of intensive care.

The development of ICU-AW has been linked to severe systemic inflammation and accelerated protein catabolism. Risk factors such as multiorgan failure, prolonged immobilization, hyperglycemia, and the administration of corticosteroids or neuromuscular blocking agents contribute to increased protein breakdown and muscle tissue injury [1]. The pathophysiology of ICU-AW can be broadly categorized into two phases: an early phase, typically within three to five days of disease onset, marked by systemic inflammation, and a late phase during which catabolic activity gradually persists [3]. Molecular mechanisms implicated in muscle degradation during the early phase include activation of calpain, the ubiquitin-proteasome system, and autophagy - a self-degradation process essential for cellular homeostasis [4]. Conversely, during the late phase, impaired or dysregulated autophagy is believed to hinder the recovery of damaged muscle tissue. Emerging evidence highlights the crucial involvement of autophagy in the progression of ICU-AW [4].

Autophagy is pivotal in immune regulation and cellular homeostasis, particularly under stress conditions such as sepsis [5]. In this context, peripheral blood mononuclear cells (PBMCs) serve as both responders and regulators of systemic inflammation through autophagic pathways, influencing immune function, mitochondrial quality control, and inflammatory signal transduction [6]. PBMCs can be collected non-invasively from critically ill patients and may reflect systemic metabolic and inflammatory responses related to muscle pathology. Recent studies suggest that reduced autophagic activity in PBMCs during sepsis may contribute to chronic inflammation and metabolic disturbances, potentially leading to muscle dysfunction and the onset of ICU-AW. However, the mechanistic links remain incompletely understood.

Accordingly, this study aimed to compare autophagic activity in PBMCs between patients with and without ICU-AW. Additionally, we sought to assess phase-specific patterns of autophagy (early vs. late phase) and their associations with immune and inflammatory markers in critically ill patients.

## Materials and methods

Study design and participants

This single-center, cross-sectional observational study was conducted in the ICU of Fujita Health University Hospital, Toyoake, Japan. The ICU is a specialized 18-bed unit that provides care for critically ill patients. Adult patients (aged 18 years or older) admitted to the ICU and requiring mechanical ventilation for more than 48 hours between April 2020 and March 2022 were eligible for inclusion. Exclusion criteria included patients with central nervous system disorders, severe musculoskeletal conditions, pre-existing movement disorders, impaired ambulation before ICU admission, confirmed COVID-19 infection, or death during the ICU stay. All included patients received standard early mobilization therapy seven days a week, administered by experienced physiotherapists according to institutional protocols [7]. Sessions included mobilization, muscle strengthening, range-of-motion exercises, and postural drainage.

Data collection

Patient data were collected from electronic medical records. They included demographic characteristics (age, sex, and body mass index), admission diagnosis, disease severity scores (APACHE II, or Acute Physiologic Assessment and Chronic Health Evaluation II and SOFA, or Sequential Organ Failure Assessment), Charlson Comorbidity Index, ICU and hospital stay length, and mechanical ventilation duration. The APACHE II score was calculated using physiological and clinical parameters obtained within the first 24 hours of ICU admission, including mechanical ventilation, vasopressors, and renal replacement therapy. Biochemical parameters were assessed from arterial blood samples collected during the early phase (within 48 hours of ICU admission) and late phase (day 7 after ICU admission).

Assessment of autophagic activity in PBMCs

PBMCs were isolated from blood samples obtained during the early and late phases. Autophagic activity was assessed using the DAPGreen Autophagy Detection Kit (Dojindo, Kumamoto, Japan), which fluoresces upon the formation of autophagosomes. The fluorescence intensity correlates with the accumulation of LC3-II, a well-established marker of autophagic activity [8]. Isolated PBMCs were incubated with 0.1 μM DAPGreen for 30 minutes at 37°C, washed twice, and then subjected to flow cytometric analysis using FACSCalibur™ (Becton Dickinson, Franklin Lakes, NJ, USA). With cellular debris excluded, PBMCs were identified by forward and side scatter gating. The detailed flow cytometry protocol and gating strategy are provided in the Appendices. Mean fluorescence intensity (MFI) of gated PBMCs was recorded and used as a quantitative index of autophagic activity [9].

Diagnosis of ICU-AW

Patients were categorized into ICU-AW and non-ICU-AW groups at the time of ICU discharge. Muscle strength was assessed using the Medical Research Council (MRC) sum score by a trained physical therapist. The MRC score, which evaluates bilateral upper and lower limb strength, was measured at least twice, with intervals of 48 hours between measurements. An MRC score of less than 48 (out of 60) was used to define ICU-AW [10].

Ethical considerations

The study was approved by the Ethics Committee of Fujita Health University Hospital (approval date: May 22, 2019; approval number: HM18-427). Informed consent was obtained from all participants or their legal surrogates. All procedures were conducted following the principles outlined in the Declaration of Helsinki.

Statistical analysis

Continuous variables were summarized as medians with interquartile range (IQR), and categorical variables were presented as frequencies or percentages. Group comparisons were performed using the Mann-Whitney U test for continuous variables and the Chi-square test for categorical variables. Statistical analyses were conducted using JMP^®^ Pro version 18.0 (SAS Institute Inc., Cary, NC, USA). A p-value of less than 0.05 was considered statistically significant.

## Results

**Figure 1:**
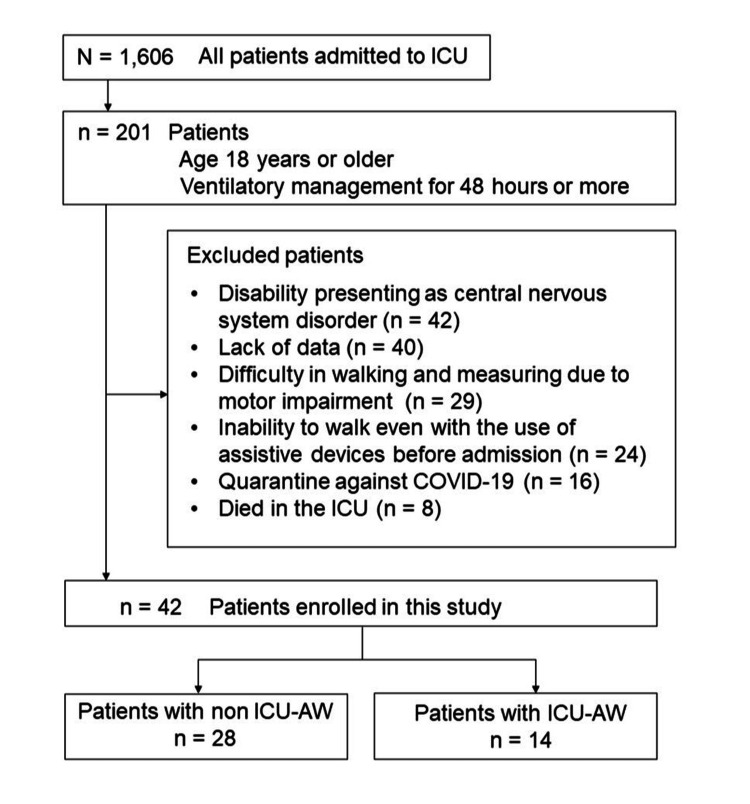
Participants flow ICU-AW, intensive care unit-acquired weakness

The participant flow is illustrated in Figure 1. Of the 201 patients screened, 42 met the inclusion criteria and were enrolled in the study (26 males; median age: 72.5 years). The clinical characteristics of the study population are summarized in Table 1.

**Table 1:** Patient characteristics of the study population Data are presented as median (interquartile range) or number (%). BMI, body mass index; ICU, intensive care unit; APACHE, acute physiologic assessment and chronic health evaluation; SOFA, sequential organ failure assessment

Variables	
Age, years	72.5 (64.5, 77.0)
Male, number (%)	26 (61.9)
BMI, kg/m^2^	24.4 (20.8, 27.3)
Length of hospital stay, days	40.9 (29.7, 64.2)
Length of ICU stay, days	6.8 (5.7, 9.4)
Length of MV, days	3.8 (2.9, 5.2)
Time to start MV, days	0 (0, 0)
Surgical patients, number (%)	25 (59.5)
Pneumonia, number (%)	8 (19.0)
Heart failure, number (%)	4 (9.5)
Acute myocardial infarction, number (%)	3 (7.1)
Others, number (%)	2 (4.8)
Sepsis shock, number (%)	12 (28.6)
Noradrenalin use, number (%)	24 (57.1)
Vasopressin use, number (%)	24 (57.1)
Systemic steroid use, number (%)	12 (28.6)
Renal replacement therapy, number (%)	9 (21.4)
Delirium incidence, number (%)	27 (64.3)
Rehabilitation days, days	40.9 (29.7, 64.2)
Time to start rehabilitation, days	0.7 (0.5, 0.9)
Time to initial sitting, days	2.2 (1.2, 3.6)
Time to initial ambulation, days	3.7 (3.2, 5.6)
APACHE Ⅱ at ICU admission, points	29 (25, 32)
SOFA score at ICU admission, points	11 (9.5, 13.5)
Maximum SOFA score in ICU, points	12 (11, 14)
Clinical frail scale, points	3 (2, 3)
Charlson comorbidity index, points	3 (2, 3)

At ICU discharge, 14 patients (33.3%) were diagnosed with ICU-AW (Table 2). Compared with the non-ICU-AW group, patients in the ICU-AW group were significantly older (ICU-AW vs. non-ICU-AW patients: 76.0 (70.2, 81.8) vs. 71.0 (58.0, 73.5) years; p = 0.008), had longer durations of mechanical ventilation (4.8 (3.1, 10.9) vs. 3.7 (2.9, 4.7) days; p = 0.043), and were more likely to have received renal replacement therapy (42.9% vs. 10.7%, p = 0.041). No significant differences were observed between the groups in terms of illness severity or the implementation of neuromuscular electrical stimulation therapy.

**Table 2:** Comparison of clinical characteristics between non-ICU-AW and ICU-AW patients Data are presented as median (interquartile range) or number (%); hyperglycemia, blood >200 mg/dL. BMI, body mass index; ICU, intensive care unit; MV, mechanical ventilation; APACHE, acute physiologic assessment and chronic health evaluation; SOFA, sequential organ failure assessment

Variables	Non-ICU-AW (n = 28)	ICU-AW (n = 14)	p-value
Age, years	71.0 (58.0, 73.5)	76.0 (70.2, 81.8)	0.008
Male, number (%)	17 (60.7)	9 (64.3)	1.000
BMI, kg/m^2^	24.5 (21.2, 26.9)	24.4 (18.3, 27.9)	0.699
Length of hospital stay, days	38.7 (29.5, 75.1)	46.7 (33.9, 54.9)	0.729
Length of ICU stay, days	6.8 (5.7, 8.8)	6.9 (5.6, 12.8)	0.873
Length of MV, days	3.7 (2.9, 4.7)	4.8 (3.1, 10.9)	0.043
Time to start MV, days	0 (0, 0)	0 (0, 0)	1.000
Sepsis shock, number (%)	4 (14.3)	8 (57.1)	0.008
Systemic steroid use, number (%)	6 (21.4)	6 (42.9)	0.169
Renal replacement therapy, number (%)	3 (10.7)	6 (42.9)	0.041
Delirium incidence, number (%)	17 (60.7)	10 (71.4)	0.734
Hyperglycemia, number (%)	21 (75)	13 (92.9)	0.233
Time to start rehabilitation, days	0.76 (0.51, 0.92)	0.67 (0.62, 0.98)	0.841
Time to initial sitting, days	2.1 (1.2, 3.7)	2.3 (1.2, 3.6)	0.879
Time to initial ambulation, days	3.8 (3.1, 5.5)	3.8 (3.3, 6.7)	0.829
APACHE Ⅱ at ICU admission, points	29 (25, 33)	29 (25, 32)	0.708
Maximum SOFA score in ICU, points	12 (11, 14)	11.5 (10, 12.8)	0.241
Clinical frail scale, points	3 (2, 3)	3 (2, 4)	0.607
Charlson comorbidity index, points	3 (2, 4)	3 (2, 3)	0.323
Neuromuscular electrical stimulation, number (%)	7 (25)	2 (14.3)	0.692

**Figure 2:**
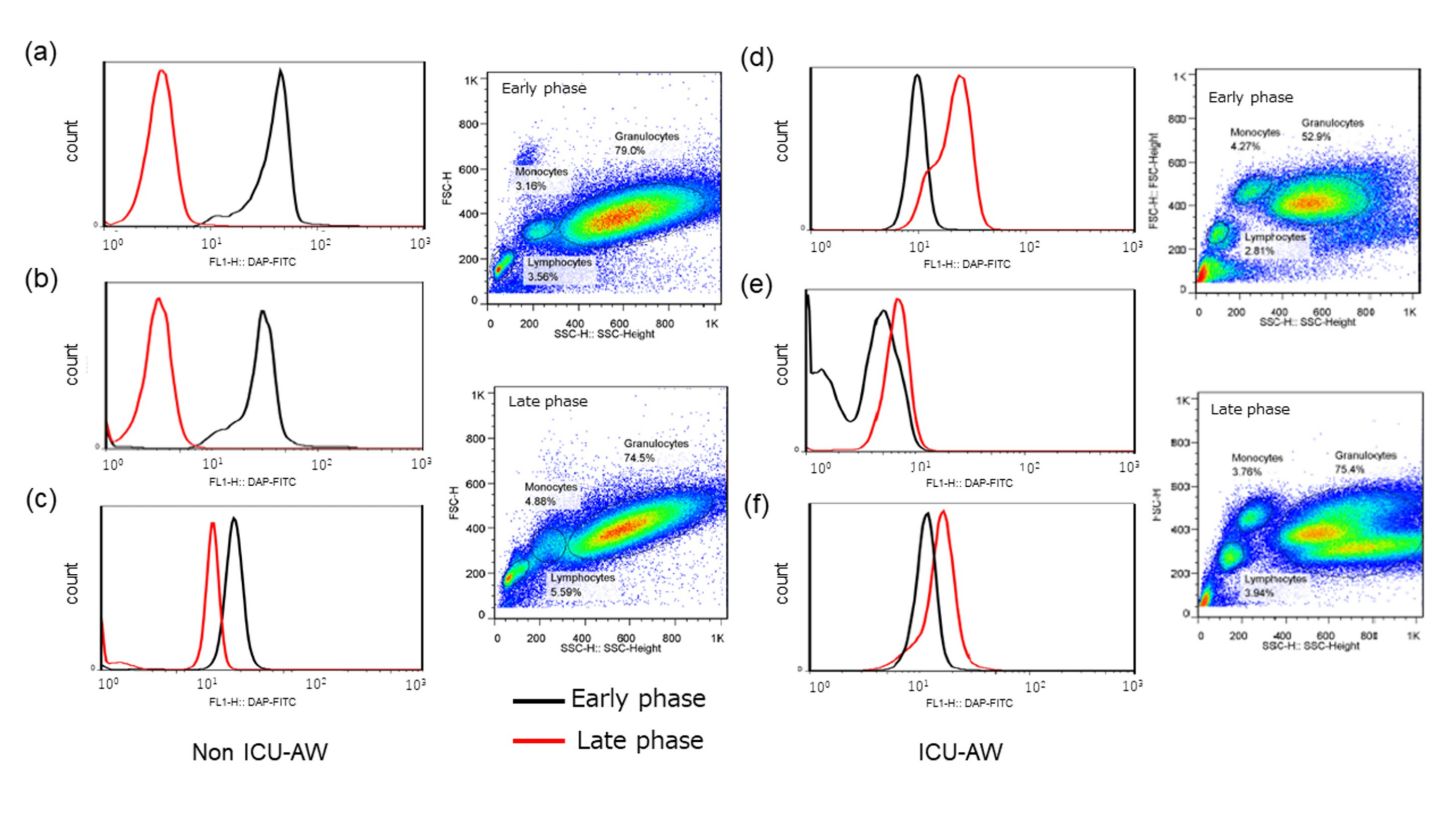
Representative histograms of PBMC subtypes Early-phase (black line) histograms for (a) granulocytes, (b) lymphocytes, and (c) monocytes in non-ICU-AW patients, showing higher fluorescence intensity; Late-phase (red line) histograms for (d) granulocytes, (e) lymphocytes, and (f) monocytes in ICU-AW patients, showing higher fluorescence intensity. PBMC, peripheral blood mononuclear cell; ICU-AW, intensive care unit-acquired weakness

Autophagic activity in PBMCs was assessed using flow cytometry. Activation was visually evaluated based on rightward shifts in histogram plots. In the ICU-AW group, 8 of 14 patients demonstrated rightward histogram shifts in the late phase (Figure 2). In contrast, in the non-ICU-AW group, rightward shifts were consistently observed during the early phase. 

PBMCs were categorized into granulocytes, lymphocytes, and monocytes. The proportion of monocytes was significantly lower in the ICU-AW group compared to the non-ICU-AW group (Table 3). Furthermore, during the late phase, the ICU-AW group showed significantly lower levels of hemoglobin (11.3 (10.6, 11.6) vs. 11.9 (11.2, 12.7) g/dL; p = 0.039) and albumin (2.4 (2.2, 2.8) vs. 2.8 (2.5, 2.9) g/dL; p = 0.007). Interleukin-6 levels tended to be higher in the ICU-AW group during the late phase (65.5 (28.7, 108.3) vs. 23.2 (13.7, 100.9) pg/mL; p = 0.076).

**Table 3:** Comparison of study markers between non-ICU-AW and ICU-AW patients Early phase: 48 hours after ICU admission; Late phase: 7 days after ICU admission. PBMCs, peripheral blood mononuclear cells; ICU-AW, intensive care unit acquired weakness; IQR, interquartile range

	Early phase							Late phase						
	Non-ICU-AW (n = 28)			ICU-AW (n = 14)				Non-ICU-AW (n = 28)			ICU-AW (n = 14)			
	Median	IQR		Median	IQR		p-value	Median	IQR		Median	IQR		p-value
Hemoglobin, g/dL	11.3	10.2	12.8	10.5	9.8	11.5	0.191	11.9	11.2	12.7	11.3	10.6	11.6	0.039
Albumin, g/dL	2.7	2.4	3.5	2.6	2.4	3.5	0.639	2.8	2.5	2.9	2.4	2.2	2.8	0.007
C-reactive protein, mg/dL	6.9	4.1	10.9	7.5	4.8	12.3	0.575	5.3	3.5	6.6	4.8	3.6	5.6	0.779
Creatinine, mg/dL	1.02	0.96	1.15	1.06	0.99	1.17	0.479	1	0.92	1.07	1.06	1.03	1.12	0.051
Blood urea nitrogen, mg/dL	26.0	23.7	29.0	27.0	24.0	29.8	0.592	26.0	24.0	28.3	28.5	26.0	31.8	0.045
Total bilirubin, mg/dL	0.8	0.2	1.4	0.8	0.9	2.7	0.980	0.8	0.4	0.9	0.6	0.2	0.8	0.383
Interleukin-6, pg/dL	136.1	55.6	407.3	189.3	114.6	808.5	0.191	23.2	13.7	100.9	65.5	28.7	108.3	0.076
% of granulocytes	62.1	51.7	75.1	72.8	62.1	74.6	0.106	54.2	48.1	63.7	66.7	51	74.5	0.076
% of lymphocytes	2.2	1.5	5.5	3.2	2.4	5.0	0.280	5.8	3.5	6.8	2.0	1.9	4.5	0.096
% of monocytes	2.2	1.5	2.9	1.5	0.8	1.8	0.034	3.1	2.3	3.9	1.5	1.4	1.8	0.013

**Figure 3:**
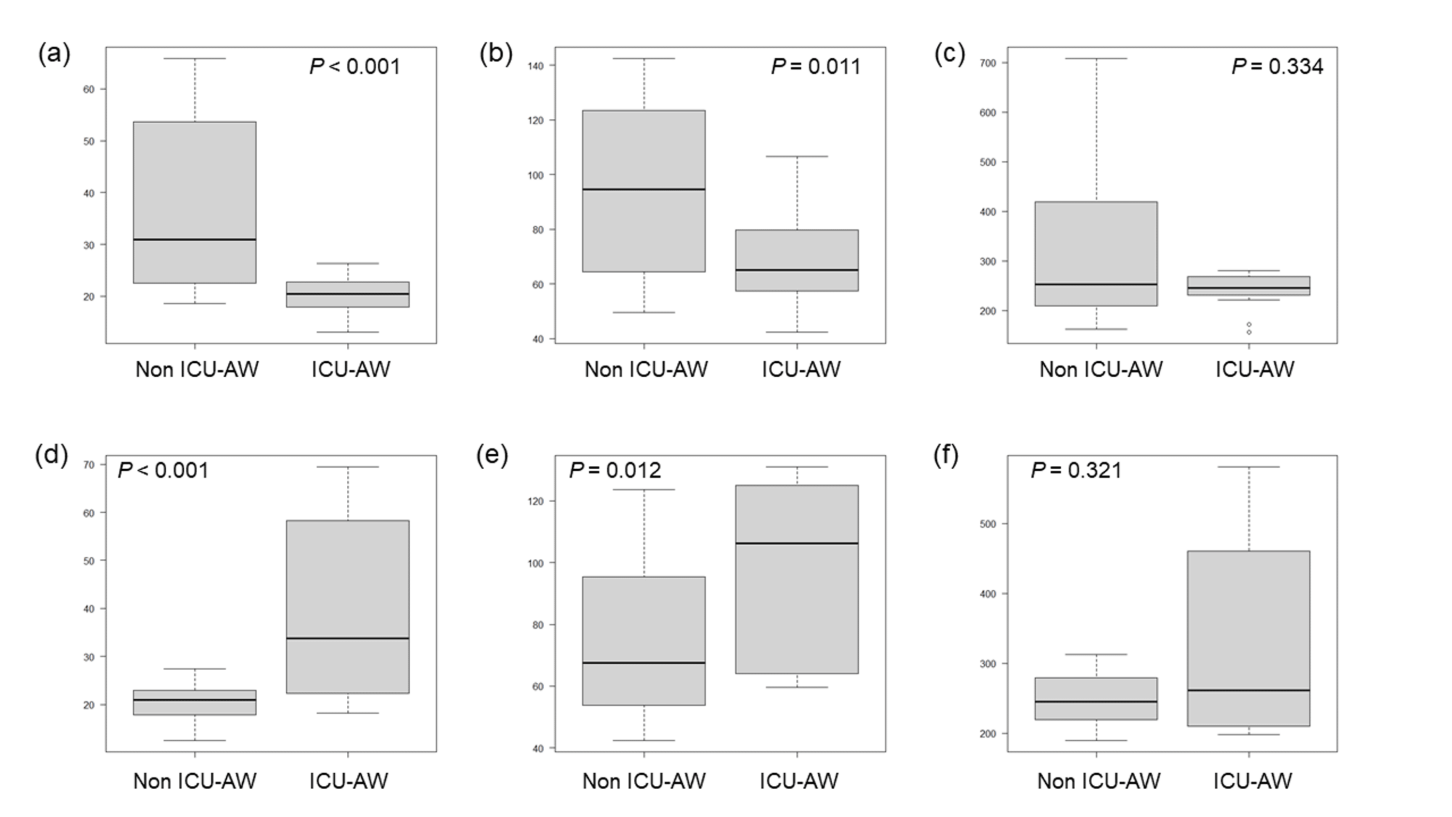
Comparison of MFI between ICU-AW and non-ICU-AW groups Early-phase MFI of (a) granulocytes, (b) lymphocytes, and (c) monocytes; Late-phase MFI of (d) granulocytes, (e) lymphocytes, and (f) monocytes. MFI, mean fluorescence intensity; ICU-AW, intensive care unit-acquired weakness

Autophagic activity in PBMCs was quantitatively analyzed based on MFI (Table 4 and Figure 3). During the early phase, MFI values of granulocytes and lymphocytes were significantly lower in the ICU-AW group than in the non-ICU-AW group. In contrast, during the late phase, MFI values of granulocytes and lymphocytes were significantly higher in the ICU-AW group. No significant difference in monocyte MFI was observed between the early and late phases.

**Table 4:** Comparison of MFI in a subset of PBMCs from non-ICU-AW and ICU-AW patients Early phase: 48 hours after ICU admission; Late phase: 7 days after ICU admission. PBMCs, peripheral blood mononuclear cells; MFI, mean fluorescent intensity; ICU-AW, intensive care unit acquired weakness; IQR, interquartile range

	Early phase							Late phase						
	Non-ICU-AW (n = 28)			ICU-AW (n = 14)				Non-ICU-AW (n = 28)			ICU-AW (n = 14)			
	Median	IQR		Median	IQR		p-value	Median	IQR		Median	IQR		p-value
Granulocytes	30.9	22.6	51.7	20.4	18.0	22.6	<0.001	21.0	17.9	22.9	33.8	22.9	56.0	<0.001
Lymphocytes	94.6	64.9	123	65.2	58.0	77.5	0.011	67.5	54.4	93.5	106.2	64.6	124.5	0.012
Monocytes	252.5	210.9	399.7	245.3	231.3	265.4	0.334	244.9	219.8	278.6	261.7	210.9	442.3	0.321

## Discussion

The findings of this study suggest that impaired autophagic activity in PBMCs during the early phase of critical illness may contribute to the development of ICU-AW. In all non-ICU-AW patients, autophagy was activated within 48 hours of ICU admission, whereas several ICU-AW patients exhibited insufficient autophagic activation during the same timeframe. Autophagy is a fundamental cellular process, responsible for the degradation and recycling of intracellular components, playing a crucial role in maintaining homeostasis and responding to cellular stress [11]. These results underscore the potential importance of immune cell-mediated homeostasis in the pathogenesis of ICU-AW.

Early-phase autophagic activity

Autophagy activation in the early phase of critical illness is believed to suppress excessive inflammation and maintain mitochondrial function. In sepsis, impaired autophagy has been linked to dysregulated inflammatory responses and increased cell death [5]. Autophagy serves as a protective mechanism through the formation of autophagosomes, which target damaged organelles and pathogens for degradation. During the hyperinflammatory phase of sepsis, autophagy is thought to facilitate pathogen clearance, neutralize toxic substances, preserve mitochondrial integrity, and regulate cytokine secretion [12]. Thus, dysfunctional or insufficient autophagy may exacerbate tissue damage and organ dysfunction.

In this study, the non-ICU-AW group exhibited activated autophagy in PBMCs during the early phase, followed by a subsequent decline in the late phase. This pattern suggests that early activation of autophagy may have mitigated inflammatory and metabolic stress, thereby contributing to cellular and tissue protection.

Furthermore, impaired autophagy in the early phase may hinder skeletal muscle recovery. A subanalysis of the EPaNIC (Early Parenteral Nutrition completing enteral nutrition In Critical illness) trial reported that patients receiving early parenteral nutrition exhibited reduced muscle strength and delayed recovery [13]. The study demonstrated that early parenteral nutrition suppressed autophagy in skeletal muscle, as indicated by lower LC3-II/LC3-I ratios. Excessive nutrient intake has been shown to suppress autophagy [14], supporting the hypothesis that impaired autophagic function contributes to ICU-AW [13]. Our findings align with these observations, as ICU-AW patients exhibited lower autophagic activity in PBMCs during the early phase.

Late-phase autophagy and ICU-AW

In this study, 8 of the 14 ICU-AW patients exhibited elevated autophagic activity in the late phase, a novel finding that suggests a delayed or compensatory autophagic response. This phenomenon may reflect either a prolonged inflammatory state or a late-phase activation of autophagy, similar to the response observed in chronic conditions such as cancer [15]. Although the precise implications remain speculative, delayed autophagic activation may contribute to persistent inflammation and impaired muscle recovery. Further studies are needed to investigate whether this subgroup represents a distinct pathophysiological mechanism within ICU-AW.

Immune function and ICU-AW

Given that PBMCs are key mediators of immune function, the relationship between autophagy and ICU-AW may be influenced by immune dysregulation. ICU-AW has been associated with systemic inflammation, multiorgan failure, and sepsis [16]. Risk factors such as older age, female sex, and organ dysfunction are also linked to impaired immune function [17]. Studies have demonstrated that severe sepsis induces simultaneous upregulation of inflammatory and immunosuppressive genes, with more severe cases exhibiting stronger immunosuppressive gene expression [18,19].

The concept of persistent inflammation, immunosuppression, and catabolism syndrome (PIICS) has been proposed to describe this pathological state [20]. Immunoparalysis following sepsis is characterized by lymphocyte apoptosis and reduced immune cell counts [21]. Research has shown that therapies targeting both inflammation and immunosuppression are critical to improving outcomes [22,23]. The clinical features of PIICS, including chronic inflammation and progressive catabolism, closely resemble those of ICU-AW [16]. In our study, ICU-AW patients exhibited higher granulocyte proportions and lower lymphocyte counts during the late phase. Previous reports have implicated impaired PBMC autophagy in immune dysfunction [24], suggesting a possible link between immune dysregulation and ICU-AW pathogenesis.

Additionally, monocyte counts were reduced in ICU-AW patients. This reduction may result from bone marrow suppression or tissue migration, commonly observed in sepsis [25]. Monocytes exhibit high autofluorescence and are sensitive to intracellular changes and inflammatory stimuli [26,27], which may affect the interpretation of fluorescence intensity data. Nevertheless, these findings reinforce the association between immune alterations and ICU-AW.

Clinical implications

This study emphasizes the importance of optimizing autophagic function during the early phase of critical illness as a potential strategy to prevent ICU-AW. Additionally, the excessive autophagic activity observed in the late phase may contribute to muscle atrophy, suggesting that phase-specific interventions may be warranted. Several studies have proposed approaches to modulate autophagy.

For example, in patients with coronary artery disease, decreased LC3 expression in PBMCs has been reported [28], implying disease-related suppression of autophagy. Conversely, aerobic exercise has enhanced autophagy in PBMCs [29]. Furthermore, early mobilization in septic patients has been reported to normalize autophagic activity [30]. These findings support the potential of early-phase rehabilitation and nutritional strategies to promote appropriate autophagic activation, preserve immune function, and maintain muscle integrity.

Nevertheless, ICU-AW is a multifactorial syndrome. In addition to autophagic dysfunction, factors such as direct inflammation-induced muscle proteolysis, microcirculatory impairment, electrophysiological abnormalities, and neuromuscular junction dysfunction may also contribute [16]. These findings highlight a potential association between dysregulated autophagy and ICU-AW, particularly during the later phases of critical illness. While our results are hypothesis-generating, further studies with larger sample sizes and mechanistic approaches are needed to determine whether modulating autophagy could serve as a therapeutic target in ICU-AW.

Limitations

This study has several limitations. First, it was conducted at a single center with a relatively small sample size, which may limit the generalizability of the findings. Second, autophagic activity was assessed using MFI, a static marker, without evaluating dynamic autophagic flux, which may limit physiological interpretation. Third, only two time points (early and late phases) were evaluated, which may not fully capture temporal dynamics. Fourth, potential confounding factors, such as nutritional management, sedation, medications (e.g., corticosteroids), and rehabilitation protocols, were not independently analyzed. Fifth, ICU-AW diagnosis was based on clinical evaluation without electrophysiological confirmation, which may affect diagnostic accuracy. Furthermore, the analysis did not distinguish between PBMC subtypes (e.g., monocytes and lymphocytes), potentially masking cell-type-specific differences in autophagy. Finally, although altered PBMC autophagy may reflect systemic immune responses, our study does not establish a mechanistic link between immune cell autophagy and skeletal muscle catabolism. Despite these limitations, the study highlights the potential utility of PBMC-based autophagy assessment in the early prediction and personalized management of ICU-AW.

## Conclusions

This study identified a temporal pattern of autophagic activity in PBMCs that was associated with the presence of ICU-AW in critically ill patients. Suppressed autophagy during the early phase may be linked to impaired immune regulation and muscle maintenance, while elevated autophagic activity in the late phase may reflect ongoing systemic inflammation and delayed recovery. Although the mechanistic relationship remains unelucidated, PBMC-based autophagy profiling may have potential as a biomarker for identifying patients at risk of ICU-AW and informing future multimodal intervention strategies, including rehabilitation and nutritional support.

## Appendices

Flow cytometry protocol for autophagic activity in peripheral blood mononuclear cell (PBMC) subsets using DAPGreen

PBMC Isolation and Storage

PBMCs were isolated immediately after venous blood collection using density gradient centrifugation with Ficoll-Paque™ PLUS (GE Healthcare, Chicago, IL, USA). The isolated cells were resuspended in RPMI-1640 medium supplemented with 10% fetal bovine serum and stored at 4°C until staining. All samples were processed on the day of collection, and no freeze-thaw cycles were applied prior to flow cytometric analysis.

DAPGreen Staining Protocol

Autophagic activity was evaluated using DAPGreen (Dojindo Molecular Technologies, Rockville, MD, USA; Cat# D676), a fluorogenic probe that accumulates in autophagosomes. Staining was conducted in accordance with the manufacturer's instructions. Briefly, PBMCs were adjusted to a concentration of 1 × 10⁶ cells/mL in phosphate-buffered saline (PBS), and the DAPGreen working solution (1×; diluted from a 500× stock) was added. Cells were incubated at 37°C for 30 minutes in the dark, followed by two washes with PBS. The stained cells were then subjected to flow cytometric analysis.

Flow Cytometric Acquisition and Gating Strategy

Flow cytometry was performed using a FACSCalibur™ flow cytometer (BD Biosciences, Franklin Lakes, NJ, USA), and data were analyzed with FlowJo™ software (BD Biosciences). PBMCs were initially identified by forward scatter (FSC) and side scatter (SSC) parameters. Subpopulations were gated based on SSC profiles as follows: (1) Granulocytes: High SSC; (2) Monocytes: Intermediate SSC; (3) Lymphocytes: Low SSC.

DAPGreen fluorescence was detected using the FITC channel (FL1). Unstained PBMCs were used as negative controls to determine fluorescence thresholds and confirm staining specificity. A minimum of 10,000 events per sample was acquired.

Data Presentation

Autophagic activity was quantified as the mean fluorescence intensity (MFI) of DAPGreen within each gated cell population (granulocytes, monocytes, and lymphocytes). FlowJo-generated histograms were used to visualize fluorescence intensity distributions across the subsets.
